# Treatment Strategies and Survival Outcomes in Breast Cancer

**DOI:** 10.3390/cancers12030735

**Published:** 2020-03-20

**Authors:** Kwok-Leung Cheung

**Affiliations:** School of Medicine, University of Nottingham, Royal Derby Hospital Centre, Derby DE22 3DT, UK; kl.cheung@nottingham.ac.uk

Treatment strategies for breast cancer are wide-ranging and often based on a multi-modality approach, depending on the stage and biology of the tumour and the acceptance and tolerance of the patient. They may include surgery, radiotherapy, and systemic therapy (endocrine therapy, chemotherapy, and targeted therapy). Advances in technologies such as oncoplastic surgery, radiation planning and delivery, and genomics, and the development of novel systemic therapy agents alongside their evaluation in ongoing clinical trials continue to strive for improvements in outcomes. In this Special Issue entitled, ‘Treatment strategies and survival outcomes in breast cancer’, a number of original research articles are included covering a diversity of studies, from pre-clinical and translational biomarker studies to clinical trials and population-based studies. They evaluated survival and other outcomes, including quality of life, in the context of pre-diagnosis (screening), as well as early and advanced stages of breast cancer.

With the established survival benefits of prophylactic mastectomy in women with BRCA genetic mutations, the procedure is increasingly being performed on the contralateral breast following diagnosis of breast cancer. Teoh et al. conducted a review of the literature which mostly consisted of retrospective studies with less than optimal data quality [1]. The evidence suggests a reduction of incidence of contralateral breast cancer following the procedure in those with ‘high risk’, notably those with BRCA genetic mutations, whereas survival benefits are uncertain. The overall benefits in other risk categories are even more doubtful. In the area of pre-invasive cancer, Sieuwerts et al. observed in cases of ductal carcinoma in situ an upregulation of APOBEC3B, which was previously seen in invasive carcinoma and known to be associated with poor prognosis, suggesting its potential role in early carcinogenesis [2]. There are two studies on screening. Heller looked at approximately 993,000 individuals using a national database, aiming to see why screening did not appear to decrease the incidence of stage IV breast cancer [3]. They found that among those diagnosed up front with stage IV disease, 37.6% had aggressive tumours as compared to 5.1% in those with stage 1 disease, suggesting that the two groups are from different populations with different tumour phenotypes. Regarding screening, over-diagnosis has been coined as the main concern. Fann et al. evaluated the 15-year adjusted cumulative survival of breast cancer in a cohort in Sweden, and noted that the majority of survivors could be attributed to cure arising from screening and subsequent treatments [4]. According to their interpretation, over-diagnosis had minimal contribution. 

For primary breast cancer, Corradini et al. analysed the oncological outcomes of 7565 cases of breast cancer in a case-controlled cohort study comparing breast conserving surgery followed by radiotherapy with mastectomy, showing that the former was associated with better recurrence control and survival, and as such recommended physicians to encourage women to receive such treatment [5]. While the findings are interesting, provocative and continue to be reassuring in terms of the efficacy of breast conserving surgery, their applications must be cautioned. The findings have not been consistently shown by randomised controlled trials and must be further investigated before a change in practice is implemented. This Special Issue also contains a few studies related to breast cancer in older patients. In a population-based registry study in the Netherlands, survival in these patients was found to be poorer when compared to their younger counterparts, and the observation was shown to be associated with a proportionately reduced use of surgery and increased use of primary endocrine therapy [6]. As discussed by the researchers, this phenomenon has been picked up in other studies and changes in treatment guidelines have since been made. While surgery is now the primary treatment of choice in this population as in the younger one, alternative treatments such as primary endocrine therapy may still be appropriate, especially in patients with competing causes of death due to significant comorbidities. Given this, and other needs to appropriately select treatments, including primary and adjuvant therapies in this challenging population, biomarker studies play a very important role in translational research. Three such biomarkers—LKB1 [7] and cytoplasmic cyclin E [8] (poor prognostic in the older (>70 years) population), and HDAC5 [9] (poor prognostic in the very young (<35 years) patients)—have been found to be associated with age. Furthermore, other conventional and emerging prognostic and predictive factors were investigated and reported in this Special Issue. Kim et al. highlighted the use of high lymph-node ratio following axillary surgery as an indicator of poor prognosis and the need for radiotherapy to the supraclavicular fossa in a retrospective study [10]. However, sentinel node biopsy has now become the standard axillary staging procedure, making the precise calculation of the ratio difficult. As a result, its potential clinical application is likely to be limited. In addition, Abdel-Fatah studied an emerging biomarker, ERCC1, a DNA excision repair protein, and noted its potential prognostic significance and ability to predict response in neoadjuvant chemotherapy [11]. In a different study using a cell line model, Gaule et al. identified the potential role of combining dasatinib and a c-Met inhibitor, in order to combat dasatinib resistance in triple negative breast cancer [12]. 

In the context of metastatic breast cancer, the Special Issue contains two pieces of work focusing on important new targeted therapies currently licensed for clinical use, CDK4/6 inhibitors and anti-HER2 therapies. Rossi et al. carried out a network meta-analysis comparing the combination use of individual CDK4/6 inhibitors with fulvestrant or an aromatase inhibitor [13]. They found that CDK4/6 inhibitors have similar efficacy when combined with an aromatase inhibitor in the first-line treatment of hormone receptor positive disease, and are superior to either endocrine agent as monotherapy, regardless of any other patient or tumour characteristics. While this may be seen as reassuring for those who are strong supporters of using such a combination despite the concerns on increased toxicity, the authors admitted the limitations of their meta-analysis, including not using actual patient data, the lack of uniformity in terms of prior use of endocrine therapy, and the fact that some trials employed non-standard fulvestrant dose (250 mg, rather than 500 mg). On the other hand, the PRAEGNANT Real-World Breast Cancer Registry study reviewed the landscape of using anti-HER2 therapies [14]. Both novel therapies (pertuzumab/trastuzumab and T-DM1) are utilised in a high proportion of HER2 positive breast cancer patients. Most patients were found to receive T-DM1 after pertuzumab/trastuzumab in a real-world setting. The Special Issue contains two other interesting studies regarding this disease stage. Keup et al. advocated a ‘comprehensive’ liquid biopsy, including both cell-free DNA mutational and circulating tumour cell transcriptional analyses, which could increase the chance of identifying actionable targets at which to direct therapeutic strategies [15]. Pelizzari identified the change in plasma LDH levels as a potential cost-effective biomarker of prognosis in the early course of systemic therapy [16]. According to the results of their study, patients who maintained elevated LDH levels after 12 weeks of first-line treatment experienced worse survival outcomes when compared to those with stable normal LDH levels, even after adjustment for other prognostic factors.

Finally, as opposed to survival outcomes, Hong et al. carried out a systematic review and meta-analysis of randomised controlled trials to investigate quality of life as another important treatment outcome for breast cancer [17]. Their work showed that exercise interventions improved quality of life and that the ‘time of session’ (longer than 45 minutes) appeared to be crucial in achieving significant improvement. 
